# Night-Light Image Restoration Method Based on Night Scattering Model for Luojia 1-01 Satellite

**DOI:** 10.3390/s19173761

**Published:** 2019-08-30

**Authors:** Lijing Bu, Zhenghui Xu, Guo Zhang, Zhengpeng Zhang

**Affiliations:** 1 School of Geomatics, Liaoning Technical University, Fuxin 123000, China; 2 State Key Laboratory of Information Engineering in Surveying, Mapping and Remote Sensing, Wuhan University, Wuhan 430079, China

**Keywords:** night-light remote sensing image, “glow” phenomenon, APSF, image restoration, atmospheric scattering

## Abstract

Aiming at solving the degradation problem of Luojia 1-01 night-light remote sensing images, the main reason for the “glow” phenomenon was analyzed. The APSF (Atmospheric Point Spread Function) template of night-light image was obtained from atmospheric source scattering. The template was used as the initial value in the regularization restoration model in this paper. Experiments were carried out using single point and regional images. The results demonstrate that the estimated APSF and restoration results of the method are better than those from other methods, and the image quality is improved after restoration.

## 1. Introduction

With the continuous development of remote sensing technology, different types of remote sensing data are becoming more and more abundant. In particular, night-time remote sensing, which can obtain the information of visible light sources on land and water with free cloud at night, has attracted much attention worldwide. The night-light data can capture information related to human activities, such as urban lighting, ship lighting, the light of oil well burning, and nature lighting, which has the unique ability to reflect human social activities [1]. Therefore, night-light remote sensing data can be used in many fields, such as monitoring socio-economic development [2] and war analysis [3]. Luojia 1-01 satellite is a scientific experimental satellite developed by Wuhan University. It has the function of night-light remote sensing and navigation enhancement, and was successfully launched on 2 June 2018. Its orbital height is 645 km, the ground pixel resolution more than 130 m, and the imaging width is 260 km. Luojia 1-01 satellite is equipped with a 4-megapixel CMOS sensor composed of 2048 × 2048 unique detectors that record weak nighttime light on Earth [4], and a GNSS(Global Navigation Satellite System) receiver for measuring and transmitting the satellite position and velocity [5]. Luojia 1-01 adopts the highly integrated design of imaging, compression, and storage. The mass of Luojia 1-01 is very light, and the rolling shutter array imaging design can last for a long time, and its noise level is low. The design with a high dynamic range greater than 96dB can meet the requirements of long exposure imaging at night. In addition, Luojia 1-01 can be used for imaging all day and night. Compared with other night-light remote sensing data, the data of Luojia 1-01 has the characteristics of high spatial resolution, high temporal resolution, high radiation resolution, and high signal to noise ratio. Thus, night-light remote sensing data of Luojia 1-01 have a wide application space in many fields such as estimating socio-economic parameters [6], evaluating artificial light pollution [7], and change detection [8]. Although Luojia 1-01 makes up for the shortcomings of the existing night-light remote sensing imaging platform in the design process, the image quality will be reduced in the process of camera imaging, under the influence of low illumination at night, optical imaging system, electronic signal conversion, space radiation [9], tremor of satellite platform [10,11], satellite viewing angles [12], atmospheric disturbance, imaging environment and other factors. Moreover, the main application field of night-light image is to estimate social and economic indicators, and the brightness value is particularly important for the study of these aspects. Therefore, under the constraints of the current satellite hardware level, and with the need to improve image quality through post-processing by using software, the main target of this paper is to restore degraded images by using image restoration. The estimation of PSF (Point Spread Function) is very important in image restoration, which can be solved by analyzing the degradation factors of images.

**Figure 1:**
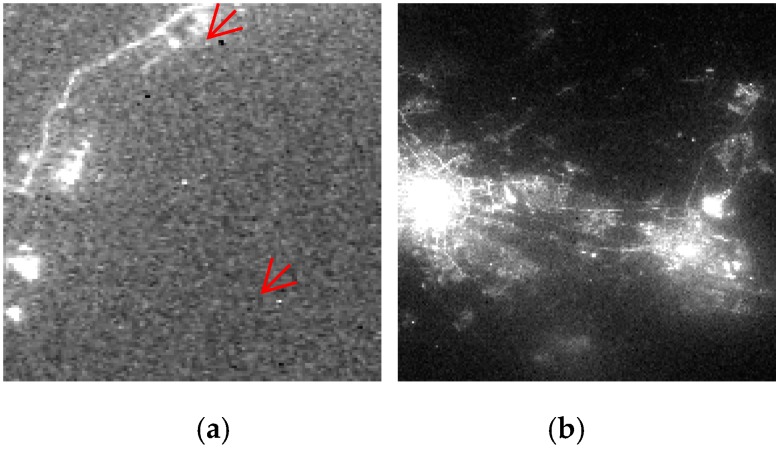
Degradation phenomenon of Luojia 1-01 night-light date: (**a**) Isolated noise degradation phenomenon in Beijing; (**b**) Comprehensive degradation phenomenon in Wuhan.

The main reasons for the quality degradation of Luojia 1-01 night-light image are as follows: First, the image degradation is caused by night imaging in a low-light photographing environment. The differences between the night-light image and the optical image taken in the daytime is that the scene being in the picture is a single one, there is no texture spectrum information and it is prone to producing noise [13], as shown in Figure 1a. Second, the image degradation is caused by the complex atmospheric environment of night imaging. Although satellite photography often uses clear and cloudless weather conditions, the effect of particles in the atmosphere on the multi-scattering of light remains significant, including due to clouds and haze. The general appearance of multiple scattering is the “glow” around the light source in various weather conditions, which is the most obvious cause of degradation for night-light images. These reasons eventually result in a comprehensive quality reduction effect, as shown in Figure 1b. Therefore, this paper focuses on the atmospheric environment during imaging, which is a major degradation factor, and studies the restoration method based on APSF estimation in the imaging environment at night.

The estimation of PSF and selection of image restoration model are main problems in image restoration. The PSF can be estimated individually or calculated at the time of recovery. As for the PSF estimation, there are many PSF estimation methods for daytime images, such as APSF estimation based on multiple scattering [14], APSF estimation based on the geometric relationship among sensors, target pixels and neighboring pixels [15]. There is also some research on APSF estimation under low illumination conditions. For example, the APSF estimation of common ground image under weather conditions such as mist, haze, and rain was studied [16,17,18]. The PSF estimation of the source degradation image was also studied [19]. There are more recovery methods during daytime or normal low illumination conditions. For example, Bayesian blind deconvolution image restoration was proposed by Karam [20], Degraded image restoration for uniform linear motion was proposed by Yang Guijun [21], constrained least squares Richardson-Lucy restoration was proposed by Richardson [22], PSF estimation and restoration of image linear features was proposed by Liu Zhengjun [23], a regularization model based on *L_p_* norm was proposed by K. Bredies [24], and a Blind deconvolution method for normalized sparse constraints was proposed by Dilip [25]. Most of these methods use the geometric or texture information of the image for PSF estimation and restoration. In general, the regularization method can effectively use the prior information in the image to reduce the sensitivity of the model to noise, and is currently widely used in the field of image processing.

Due to the low spatial resolution of most commonly used night-light remote sensing data (such as DMSP-OLS, NPP-VIISR), the image lacks necessary details. At present, there is little research on image restoration of night-light remote sensing data. The Luojia 1-01 night-light remote sensing image has a higher spatial resolution, and the image brightness details are increased. The identifiable approximate point features are obvious, such as independent ships or large landmark buildings. These isolated point sources provide good data conditions for APSF estimation of night-light images. The night-light image data of Luojia 1-01 has been corrected by radiation and the image quality has been improved. However, radiometric correction only eliminates various distortions attached to the radiance in the image data [26]. The “glow” phenomenon caused by atmospheric scattering of nighttime lights belongs to the degradation problem in the image, which cannot be eliminated by radiometric correction, although the image quality can be further improved by using image restoration. For the current application of night-light remote sensing data, in addition to radiometric correction and other necessary processing, denoising and other processing should be carried out [27]. According to the image degradation and restoration model, restoration can achieve the effect of denoising and improving quality. Therefore, it can also provide more accurate data as a basis for estimating socio-economic indicators.

In this paper, the APSF estimation method and ratio sparse constraint restoration model were proposed aiming at problems of the fuzzy quality reduction and noise in night-light images of Luojia 1-01. In the problem of APSF estimation, the point object with better imaging quality in the image was selected, and the initial value of APSF was obtained according to the atmospheric transfer equation. Generally, the initial value of APSF estimated in this way may be accurate around the point object, but because the value will be different far away from the point, it means the initial value of APSF estimated cannot properly reflect the APSF of the whole image [28]. In order to get the best APSF for the whole image, the initial value needs to be further optimized. There is also some noise in night-light images, so we proposed to further optimize the APSF value by adopting the ratio sparse constraint restoration model and reduce the noise in images.

## 2. Degradation Model of Night-Light Image and Atmospheric Scattering Analysis

If the degradation function is a linear and spatially invariant process, the degradation model of night-light remote sensing images is shown in Equation (1) [29].

(1) g(u,v)=f(u,v)⊗h(u,v)+n(u,v)

where, g(u,v) represents degraded images of observations, f(u,v) is the original clear image, h(u,v) is the image degradation matrix, ⊗ stands for convolution, n(u,v) is noise. The image degradation discussed in this paper is only related to an atmospheric imaging environment.

**Figure 2:**
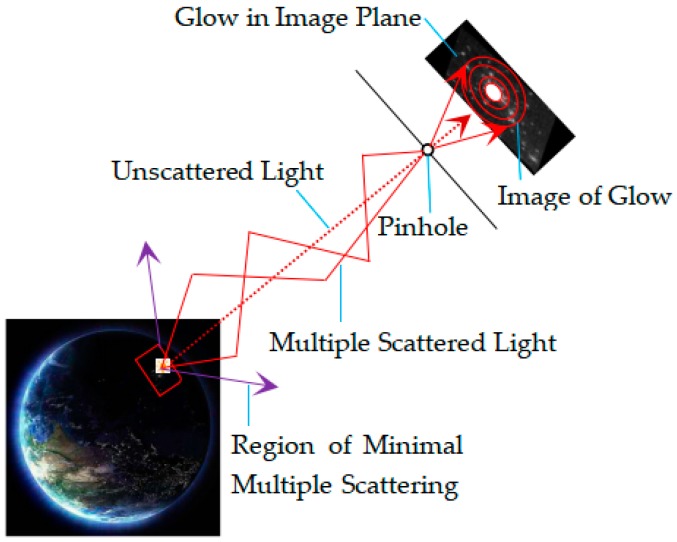
Multiple scattering glow model of light from a source to a sensor.

In the ideal state, the light propagates along a straight line in the atmosphere without deviation in the direction, and shines directly through the pinhole to the imaging plane, which is called the “ideal imaging point”. However, in the actual situation, imaging environments such as the atmosphere, weather conditions, and clouds have an effect on the image. Because the particles in the atmosphere have an effect on the light, the light will be propagated towards a different direction. The light from the light source gets scattered multiple times in different directions through the atmosphere. As a result, the light will diffuse to the periphery of the “ideal imaging point” when passing through the pinhole, resulting in the phenomenon of “glow”. For night-light remote sensing images, the range of the glow phenomenon caused by atmospheric scattering at night is different from that photographed on the ground. Figure 2 shows the influence of atmosphere scattering on the imaging of the light source at night. The light from the light sources on the ground propagates in the atmosphere. Due to the influence of particles in the atmosphere, the received light includes multiple scattering and no scattering, and the range of the “glow” phenomenon will be different because the photographing position and height are different from the ground.

The effect of light sources interaction is different between night-light remote sensing images and common ground night images. The orbital altitude of Luojia 1-01 is 645 km, and the imaging distance is long. The photographing angle of the satellite can be generally regarded as vertical ground photographing, and the influence of the shape and type of light source is also smaller. A single pixel of an image with a spatial resolution of 130 m captures the sum of light sources information within the range of 130 m × 130 m, so the night-light image captures the comprehensive brightness value of multiple light sources. Moreover, the range of field of view produced is smaller than that of common ground photographing at night, and the intensity of the light source in most directions is cut off, resulting in a relatively small scattering scale. As such, the range of “glow” produced by satellite images is small, as shown in Figure 2. The ground night scene usually has a variety of active light sources, such as street lights, car lights, house building lights, and so on, while the imaging distance is short, usually within a range of several meters to several hundred meters. These sources interact with each other due to their relative position, strength, type, and shape, resulting in a brighter image than the actual natural atmospheric light and a larger “glow” range. Image restoration of common ground night images recover the information of buildings and people. However, image restoration of night-light remote sensing images recovers the true brightness information. All the above factors will affect the estimation of atmospheric point spread function.

## 3. APSF Estimation Model of Luojia 1-01

**Figure 3:**
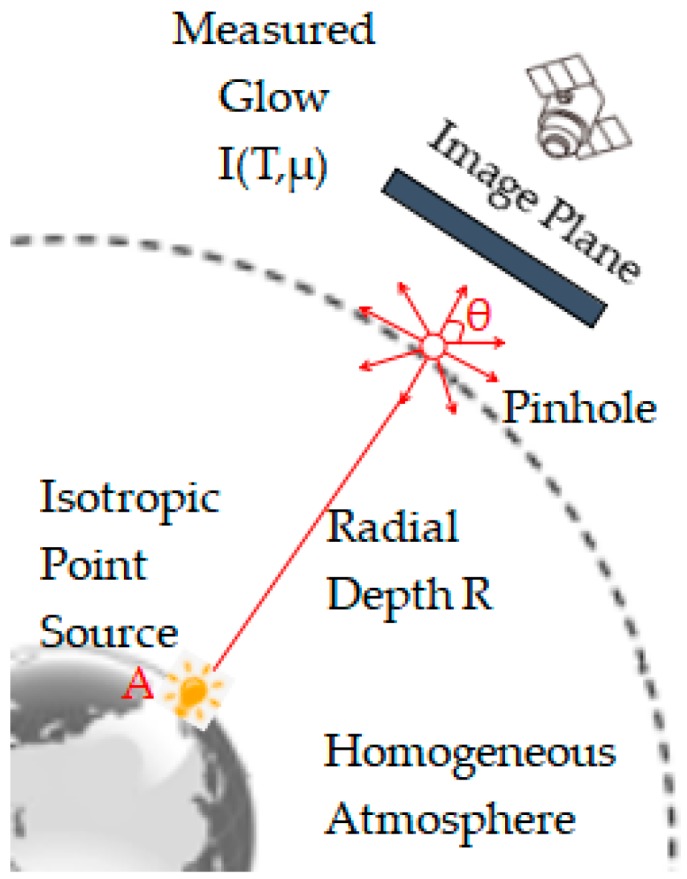
Scattering of an isotropic point light source in homogeneous atmosphere.

This paper analyzes the scattering characteristics of the point source at night in the atmosphere which is homogeneous and infinite to an extent, and estimates the shape of APSF of Luojia 1-01. In Figure 3, A represents an isolated point light source on the ground, and the atmospheric environment around it is homogeneous and isotropic. The distance between the camera and the point light source is the radiation depth R. *θ* represents the scattering angle of light. When the camera is imaging through the pinhole, it will produce “glow” on the image plane, it reflects the point diffusion effect caused by the scattering of an isolated point light source through the atmosphere. The “glow” phenomenon can reveal the effect of the atmosphere on the image. As long as we find an approximate point ground object with good image quality from the image, the APSF from night degraded image can be obtained.

APSF models under various weather conditions such as air, small aerosols, haze, fog and rain were deduced in reference [19]. According to reference [19] and the imaging conditions of Luojia 1-01, the APSF model of night-light remote sensing image is shown in Equations (2)–(5).

(2) $$I\left(T,\mu \right)=\sum_{m=0}^{\infty }\left(g_{m}\left(T\right)+g_{m+1}\left(T\right)\right)L_{m}\left(\mu \right)$$

(3) $$g_{m}\left(T\right)=I_{0}e^{-\beta_{m}T-\alpha_{m}logT}$$

(4) $$\alpha_{m}=m+1$$

(5) $$\beta_{m}=\frac{2m+1}{m}\left(1-q^{m-1}\right)$$

where, I(T,μ) is the APSF derived from the model, *m* is the number of Legendre polynomial expansion terms, when *m* = 0, g_0_ = 0, g*_m_*(T) refers to light attenuation in different weather environments, $L_{m}\mu$ is the Legendre polynomial of order *m*, it represents the diffusion of light due to scattering, I_0_ refers to the intensity of the light source, $\alpha_{m}$ and $\beta_{m}$ are coefficients, *q* is the forward scattering coefficient, and *T* is the atmospheric optical thickness, while the atmospheric optical thickness T at night can be calculated using Equation (6) [19].

**Figure 4:**
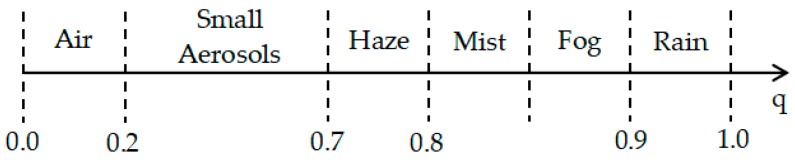
The range of forward scattering coefficient q under different weather conditions [19].

It can be seen from Equation (2) that the main influencing factors of the nighttime image APSF are the light attenuation and light diffusion in the weather environment. According to Equations (3)–(5), the essential influencing factors of $g_{m}\left(T\right)$ include I_0_, *T*, *q* and *m*. In the image with a spatial resolution of 130 m, large isolated ships at sea can be considered as a point light source, and brightness information of ship is used as value of I_0_. The value of m determines the number of Legendre polynomials to expand. *q* is generally in the range of 0 to 1, and the value of *q* is different under different weather conditions (Figure 4). Night-light image imaging is generally used in a clear night, and the value of *q* can range from 0 to 0.7.

(6) $$T=\sigma R\approx \frac{3.912}{V}R$$

where σ is the extinction coefficient of atmosphere, *R* can refer to radial depth, while T can also be shown by visual distance *V* and radial depth *R*. When we estimate APSF, *T* is less than 1, then the coefficient *m* is greater than 10, otherwise *m* is less than 10. If *T* becomes bigger, then m becomes smaller.

The essential factor affecting the light diffusion $L_{m}\left(\mu \right)$ is scattering angle *θ*, as shown in Equation (7).

(7) μ=cosθ

The APSF obtained by using Equation (2) also has the following features: (1) The APSF template is non-negative, meaning when the estimated value is less than 0, it is replaced with 0. (2) The value of APSF is less than 1, and the sum of all its elements is approximately 1 [30]. (3) The size of the APSF increases with the brightness of the light source. The brighter the light source, the greater the effect of blurring.

## 4. Image Restoration Method of Luojia 1-01 Based on Night Scattering Model

### 4.1. Image Restoration Model of Night-Light Remote Sensing

In the problem of image restoration, the regularization method has the characteristics of introducing target priori information, strong denoising ability, and high convergence stability, so it has many related and useful applications. Night-light image information mainly includes light information and black background information, and restoration is mainly to reduce degradation of the image and remove image noise. From the analysis in Section 2, it can be determined that the night-light image is taken with the light intensity, and the light will produce “glow” phenomenon when it hits images. APSF at an imaging time can be obtained by analyzing the approximately ideal isolated point light source, and this prior information can improve the “glow” phenomenon in the whole image. And according to Section 1, the initial value needs to be further optimized in order to obtain the best APSF for the whole image. 

In terms of restoration methods, blind deconvolution method for normalized sparse constraints was proposed [25]. But the method is insensitive to noise, and the model solving is complex and slow. So L_0.5_/L_2_ norm is used as the sparse constraint term to maintain information such as the boundary of lights in the image. The L_1_ norm was also added to the recovery model to increase the effect of the shape of APSF on the recovery results. In addition, the initial APSF value obtained in Section 3 was optimized. There are good sparse L_1_ and L_0_ norm. But there is also the NP-hard (non-deterministic polynomial) problem for L_0_ norm to solve. This means it is difficult to optimize. Generally, the constrained solution of the L_1_ norm is equivalent to the L_0_ norm. So L_1_ norm can be used to replace L_0_ norm as the solution of sparse constraint term for images. And L_1_ norm has the effect of suppressing noise, which can weaken image noise. So our method can accelerate the solving speed and suppress the noise in the image. The restoration model of this paper is shown in Equation (8).

(8) $$J=\underset{x,k}{\min}\gamma \|y-k⊗x{\|}_{2}^{2}+\frac{\|x{\|}_{0.5}}{\|x{\|}_{2}}+\lambda \|k{\|}_{1}$$

where, *x* is the clear image, *y* refers to the degraded image and *k* stands for the APSF of the image. $\|y-k⊗x{\|}_{2}^{2}$ is fidelity term, in order to ensure the minimum training error between the data and the original data in the calculation process. The second term is the L_0.5_/L_2_ regularization term, which is a sparse metric constraint that promotes the size of x that do not change, the third term is the APSF constraint term, the two terms guarantee that the test error of the model is small. γ is the weight value of the fidelity term, λ is the weight value of the APSF regularization term. For noisy images, as γ becomes larger, the effect of noise suppression will be stronger and the image will be smoother. As γ becomes smaller, the details of the image improve. But if γ becomes too small, it can lead to over sharpening. As λ increases, the noise in APSF is suppressed. The initial value of k is the APSF estimated in Section 3.

### 4.2. Model Solution

It is difficult to converge at the same time for the unknown variables x and k in Equation (8). However, it is easy to produce a minimum value in the local. After solving by using the method of alternating minimization [31], Equation (8) can be divided into two relatively simple and independent problems: Equations (9) and (10). The method of iterative shrinkage-thresholding method [32] and iterative weighted least square [33] are used to alternately iterate the image and *k*.

(9) $$J\left(x\right)=\underset{x}{\min}\gamma \|y-k⊗x{\|}_{2}^{2}+\frac{\|x{\|}_{0.5}}{\|x{\|}_{2}}$$

(10) $$J\left(k\right)=\underset{x}{\min}\gamma \|y-k⊗x{\|}_{2}^{2}+\lambda \|k{\|}_{1}$$

In Equation (9), $\|x{\|}_{0.5}$/$\|x{\|}_{2}$ is non-convex and cannot be solved directly. In the solution process, Equation (9) can be transformed into a convex optimization problem of L_1_ norm, two loops of inside and outside can be set. First, $\|x{\|}_{2}$ can be solved by fixed $\|x{\|}_{0.5}$. Then $\|x{\|}_{0.5}$ is used to solve $\|x{\|}_{2}$. The stopping condition of *x* is determined by Equation (11).

(11) $$\frac{t\left(x^{\left(i+1\right)}\right)}{t\left(x^{\left(i\right)}\right)}>n$$

where, $t\left(x^{\left(i+1\right)}\right)$ and $t\left(x^{\left(i\right)}\right)$ are the cost function values of the last time and the previous time, respectively, *n* is a minimum value. Avoiding the *L_p_* norm means not being guided at zero when calculating Equation (9). We can make a smooth approximation of the *L_p_* norm first [34], as shown in Equation (12).

(12) $$\left\|x{\|}_{P}^{P}=\sum_{i=1}^{M\times N}{\left|x_{i}\right|}^{P}\approx \sum_{i=1}^{M\times N}{\left({\left|x_{i}\right|}^{2}+\varepsilon \right)}^{\frac{P}{2}}\right)$$

where, ε>0 refers to minimal constant, usually $\varepsilon =10^{-5}$, *x* is a dimensional column vector of MN×1. The general form of *x* is $\left(x_{1},x_{2},x_{3},\ldots ,x_{M\times N}\right)$, and $\sum_{i=1}^{M\times N}{\left|x_{i}\right|}^{2}=x_{1}^{2}+x_{2}^{2}+x_{3}^{2}+\ldots +x_{M\times N}^{2}$. So, Equation (9) can be change into Equation (13). We can take the partial derivative of Equation (13) with respect to *x* and Equation (14) can be obtained. The solution of Equation (14) is iterated by the steepest descent method. Finally, the optimal *k* is solved by Equation (10).

(13) $$J\left(x\right)=min\gamma \|y-k⊗x{\|}_{2}^{2}+\sum_{i=1}^{M\times N}{\left({\left|x_{i}\right|}^{2}+\varepsilon \right)}^{\frac{P}{2}}$$

$$\frac{\partial J\left(x\right)}{\partial x}=-2\gamma {\left(k\right)}^{T}\left(y-kx\right)+P\left[\sum_{i=1}^{M\times N}{\left({\left|x_{i}\right|}^{2}+\varepsilon \right)}^{\frac{P}{2}-1}\right]\cdot x$$

(14) $$=-2\gamma {\left(k\right)}^{T}\left(y-kx\right)+P\cdot diag\left[\sum_{i=1}^{M\times N}{\left({\left|x_{i}\right|}^{2}+\varepsilon \right)}^{\frac{P}{2}-1}\right]\cdot x$$

**Figure 5:**
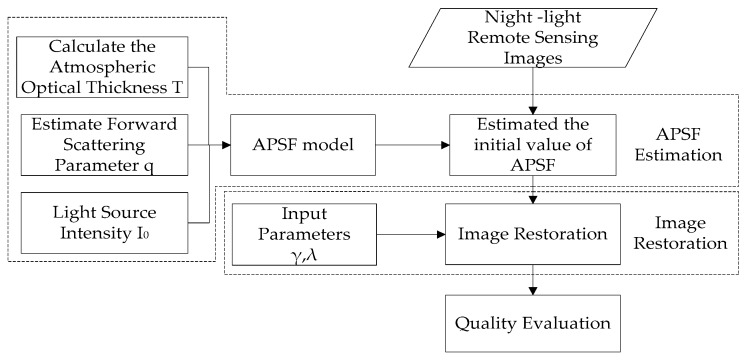
Flow chart of image restoration of night-light remote sensing.

The recovery process of night-light remote sensing image is shown in Figure 5. The detailed steps of the experiment are as follows:

1. Input the night-light remote sensing images taken by Luojia 1-01 satellite.
2. Estimate the APSF of the image. The atmospheric optical thickness *T* is calculated, the forward scattering coefficient *q* is estimated according to the weather condition, and put *T*, *q* and the light source intensity I_0_ into the atmospheric point spread function model to calculate the APSF value of the image.
3. Put APSF into the restoration model, and input parameters γ and λ, then perform image restoration with the Equation (8).
4. Output the restored image and evaluate its quality.

For image quality evaluation, the image with more detailed information and less “glow” phenomenon is better in vision. Variance and TenenGrad function were used for quantitative evaluation [35]. Variance refers to the degree of dispersion of image pixel gray values relative to the mean. If the variance is larger, it indicates that the gray value of the image is dispersed, and the image quality is better. The Tenengrad function is an image evaluation index that measures image sharpness and edge information. The larger the Tenengrad function value, the better the image quality.

## 5. APSF Estimation and Image Restoration Experiments in This Paper

### 5.1. Introduction of Experimental Data

**Figure 6:**
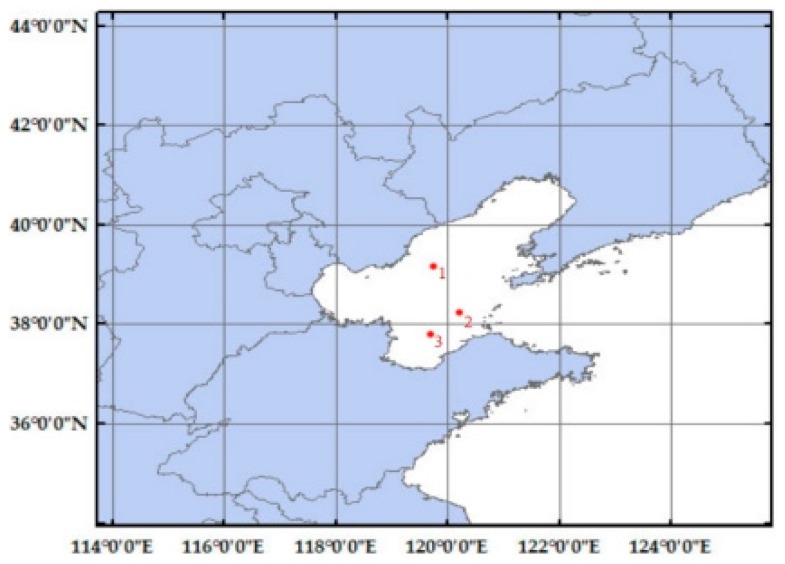
Point light distribution.

**Figure 7:**
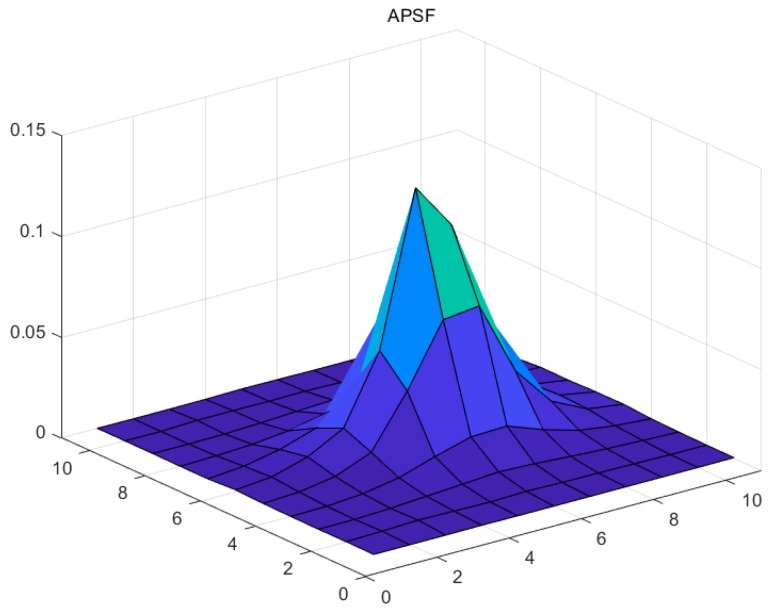
3D results.

**Figure 8:**
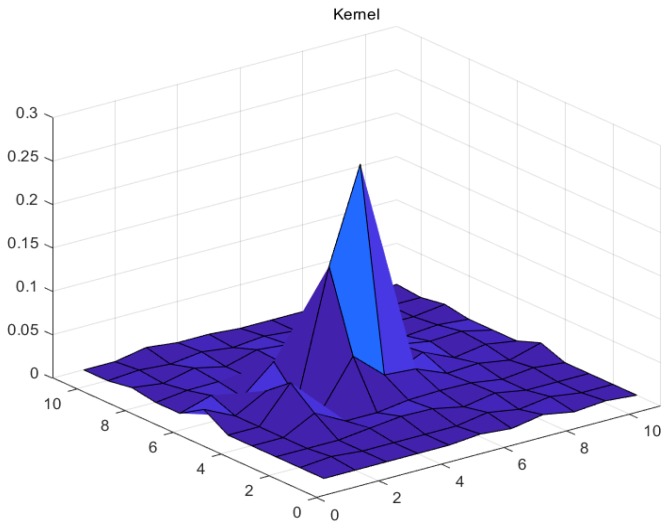
3D results of restored APSF.

This paper uses the night-light remote sensing images taken by Luojia 1-01 on 29 October 2018 as the experimental data. Three isolated point light sources with good quality in the Bohai sea (Figure 6) and night-light remote sensing images from Dongying in Shandong province and Tianjin were selected as the experimental areas. The characteristics of the experimental areas are shown in Table 1. The forward scattering coefficient *q* in the APSF estimation is 0.2, and the atmospheric optical thickness *T* is 1.2. The estimated APSF template is shown in Table 2, and the 3D result is shown in Figure 7. After restoration, the APSF template is shown in Table 3, and the 3D result is shown in Figure 8. The APSF template estimated by the method presented in this paper is estimated by analyzing the imaging blurred factors of night-light remote sensing images. It is obtained according to its own image information and imaging characteristics. The template can better reflect the characteristics of the image itself.

### 5.2. Results and Analysis

**Figure 9:**
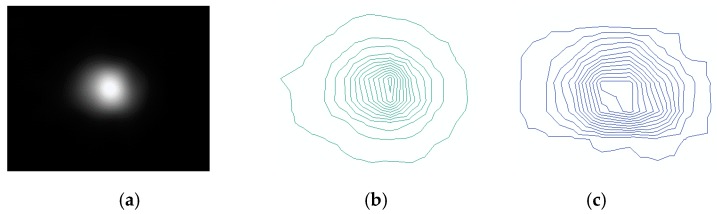
(**a**) The point source and its surrounding glow phenomenon; (**b**) Approximate representation of the blurred point light source; (**c**) Approximate representation of the point light source after restoration.

**Figure 10:**
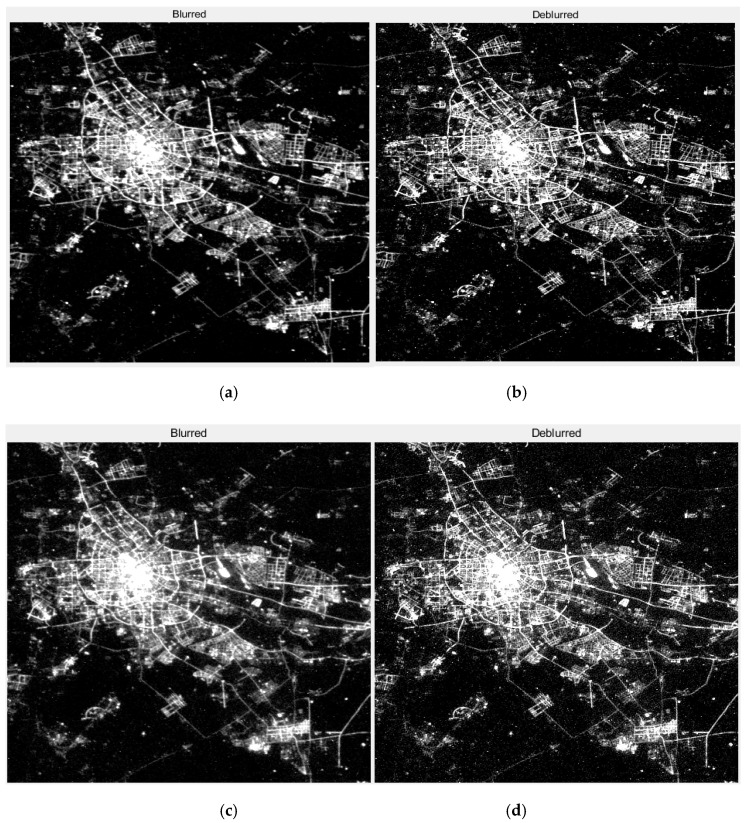
The restoration results of Tianjin in clear and hazy nights. (**a**) The original image of the clear night; (**b**) The restoration result of (**a**), T = 1.2, q = 0.2; (**c**) The original image of the hazy night; (**d**) The restoration result of (**c**), T = 4, q = 0.75.

The “glow” phenomenon around the point source is seen as a concentric circle. The image before and after restoration with this method is shown in Figure 9. When comparing Figure 9c with Figure 9b, the maximum brightness value range of the pixels is increased, the contour line is more dense, and the diffusion range is obviously contracted, which indicates that the point light source is brighter and the blurred effect is weakened after the restoration. The restoration results of night-light remote sensing data in clear and haze weather from Tianjin for different nights were compared in Figure 10. The experimental results show that the method used by this paper has a good effect on dehazing. The effect of haze in the restoration results was decreased and the clearness of the image was increased.

**Figure 11:**
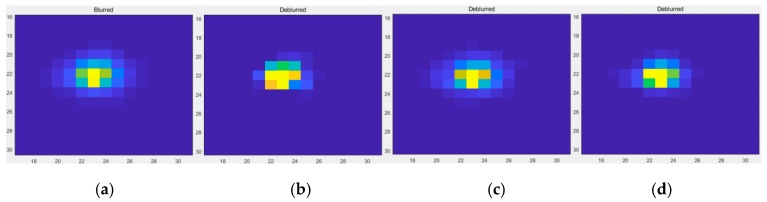
The restoration results of Point 1. (**a**) The original image; (**b**) The method used by this paper; (**c**) Method Two; (**d**) Method Three.

**Figure 12:**
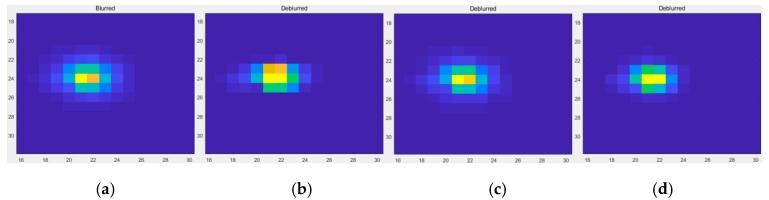
The restoration results of Point 2. (**a**) The original image; (**b**) The method used by this paper; (**c**) Method Two; (**d**) Method Three.

**Figure 13:**
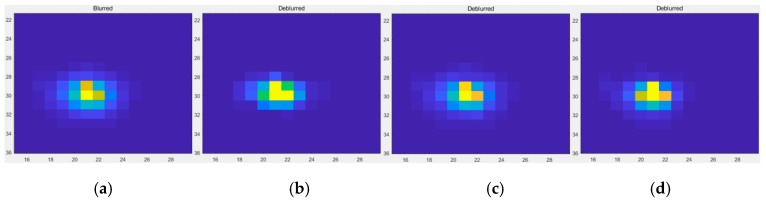
The restoration results of Point 3. (**a**) The original image; (**b**) The method used by this paper; (**c**) Method Two; (**d**) Method Three.

**Figure 14:**
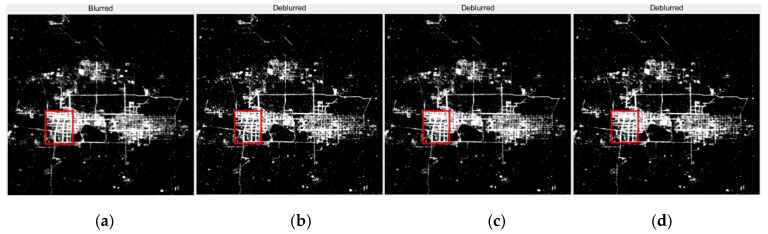
The restoration results of Dongying. (**a**) The original image; (**b**) The method used by this paper; (**c**) Method Two; (**d**) Method Three.

**Figure 15:**
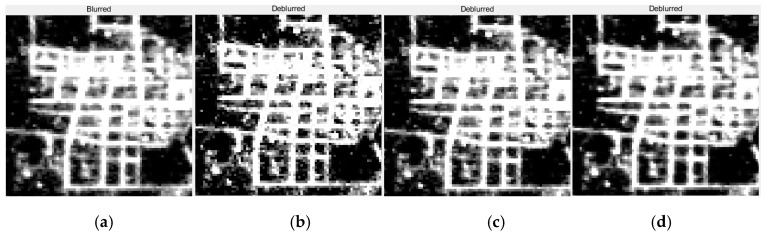
The detail restoration results of Dongying. (**a**) The original image; (**b**) The method used by this paper; (**c**) Method Two; (**d**) Method Three.

**Figure 16:**
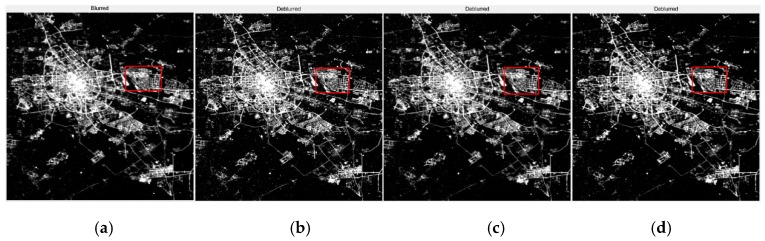
The restoration results of Tianjin. (**a**) The original image; (**b**) The method used by this paper; (**c**) Method Two; (**d**) Method Three.

**Figure 17:**
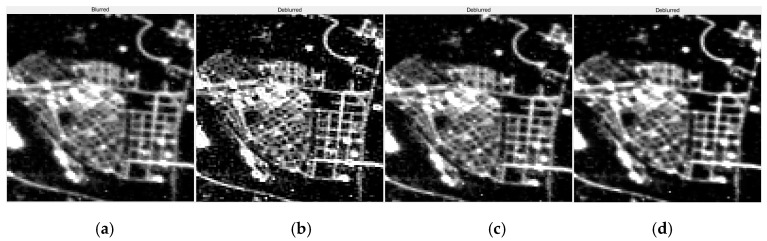
The detail restoration results of Tianjin. (**a**) The original image; (**b**) The method of this paper; (**c**) Method Two; (**d**) Method Three.

In order to further verify the effectiveness of the APSF estimation and the night-light image restoration method experiments, the blind deconvolution model was used to perform the image restoration experiment and then compared with the restoration effect of the Gaussian PSF template. The blind deconvolution model with Gaussian PSF template used to perform an experiment of image restoration. This is Method Two. The blind deconvolution model with our APSF template used to perform experiment of image restoration, which is Method Three. The experiment results are shown in Figure 11, Figure 12, Figure 13, Figure 14, Figure 15, Figure 16 and Figure 17. At the same time, the experimental results were analyzed by using image variance (Table 4) and The Tenengrad function (Table 5). 

By observing Figure 11, Figure 12 and Figure 13, it is found that compared with the original image data, in the point source recovery image obtained by the three restoration methods, the blurred effect of the point source is significantly reduced, and the brightness value is increased, which proves the effectiveness of the restoration result. At the same time, the restoration result of our method is visually superior to the Method Two and the Method Three, and the visual effect of the Method Three is better than that of Method Two. It is further illustrated that the APSF template estimated in this paper is superior to the Gaussian PSF template. At the same time, it also shows that the recovery method of this paper is more effective.

By observing Figure 14, Figure 15, Figure 16 and Figure 17, it is found that compared with the original image data, in the city recovery image obtained by the three methods has different degrees of image clarity, the noise is suppressed, the restoration result is brighter and clearer, and the image details are increased. The blurred effect has been reduced to varying degrees. Compared with Method Two and Method Three, the image details of the image after restoration are obviously increased, and the “glow” phenomenon is obviously improved, indicating that the method is effective.

By observing the evaluation of the two indicators in Table 4 and Table 5, the two indexes of the three groups of recovery results are higher than the original images, indicating that the three groups of restoration methods can improve the image quality. The recovery results of our method are higher than those of Method Two and Method Three, and the index of Method Three is better than that of Method Two.

Therefore, from the perspective of restoration effects and objective evaluation indicators, the visual evaluation and evaluation indicators of our method are superior to other methods, indicating that the image quality after restoration is improved, and the method is feasible.

## 6. Conclusions

Aiming at the “glow” phenomenon in the image, the characteristics of the Luojia 1-01 night-light image were analyzed. By analyzing the APSF parameters during night imaging, the APSF template was obtained, and the Luojia 1-01 night-light image restoration method based on the night scattering model was proposed. The images of single point and regional were used for the restoration experiment. The experimental results demonstrate that the method of the APSF estimation is feasible. The image “glow” phenomenon is improved after restoration with the method, the noise is reduced and the detail information is increased. The method used in this paper is applicable to images after radiometric correction, and can be used to improve the pre-processing stage of image quality. The processing results are helpful to improve the accuracy of the later index estimation. This method is of great significance to improve the quality of night-light remote sensing images and expand its application field.

## Figures and Tables

**Table 1:** Characteristics of experimental areas.

Experimental Areas	Imaging Time (UTC)	Weather Condition	Positioning Accuracy/m	Spatial Resolution/m	Characteristics of Experimental Areas
Three isolated point light sources	2018.10.29 14:17:34	Clear	82	130	The image quality of point light source is good, clear and bright, without interference from other light sources.
Dongying	2018.10.29 14:17:34	Clear	82	130	Geography plays an important role in the Bohai economic zone.
Tianjin	2018.10.29 14:17:34	Clear	82	130	The largest open coastal city in northern China, with a large population, plays an important geographical, economic and social role.
	2018.09.26 14:12:10	Haze	399		

**Table 2:** The APSF template estimated.

0.0000	0.0000	0.0000	0.0001	0.0000	0.0000	0.0000	0.0000	0.0000	0.0000	0.0001
0.0001	0.0005	0.0012	0.0009	0.0006	0.0011	0.0007	0.0006	0.0004	0.0001	0.0001
0.0000	0.0001	0.0008	0.0020	0.0032	0.0045	0.0038	0.0026	0.0016	0.0009	0.0003
0.0001	0.0004	0.0018	0.0049	0.0106	0.0156	0.0162	0.0096	0.0045	0.0020	0.0007
0.0003	0.0012	0.0041	0.0113	0.0369	0.0675	0.0695	0.0326	0.0127	0.0041	0.0016
0.0008	0.0024	0.0076	0.0170	0.0508	0.1267	0.1035	0.0539	0.0165	0.0047	0.0015
0.0000	0.0015	0.0050	0.0108	0.0194	0.0646	0.0581	0.0331	0.0101	0.0038	0.0011
0.0000	0.0004	0.0013	0.0032	0.0069	0.0113	0.0126	0.0095	0.0041	0.0021	0.0012
0.0001	0.0000	0.0002	0.0009	0.0018	0.0027	0.0029	0.0026	0.0016	0.0008	0.0001
0.0001	0.0000	0.0007	0.0007	0.0006	0.0008	0.0004	0.0004	0.0005	0.0006	0.0001
0.0001	0.0000	0.0000	0.0001	0.0004	0.0003	0.0001	0.0001	0.0004	0.0001	0.0000

**Table 3:** Restored APSF template.

0.0000	0.0000	0.0000	0.0000	0.0000	0.0032	0.0000	0.0064	0.0000	0.0031	0.0000
0.0000	0.0032	0.0000	0.0000	0.0000	0.0000	0.0060	0.0000	0.0051	0.0000	0.0000
0.0000	0.0000	0.0000	0.0042	0.0082	0.0074	0.0000	0.0063	0.0000	0.0000	0.0000
0.0000	0.0000	0.0185	0.0000	0.0000	0.0050	0.0062	0.0055	0.0000	0.0038	0.0000
0.0000	0.0000	0.0411	0.0000	0.0524	0.0213	0.0186	0.0040	0.0059	0.0000	0.0103
0.0170	0.0000	0.0193	0.0000	0.1434	0.2516	0.0000	0.0157	0.0000	0.0057	0.0000
0.0000	0.0110	0.0000	0.0307	0.0160	0.1125	0.0000	0.0123	0.0063	0.0083	0.0000
0.0000	0.0044	0.0049	0.0000	0.0080	0.0000	0.0101	0.0000	0.0000	0.0031	0.0000
0.0046	0.0000	0.0080	0.0000	0.0099	0.0064	0.0059	0.0061	0.0000	0.0000	0.0046
0.0000	0.0028	0.0000	0.0045	0.0000	0.0035	0.0000	0.0000	0.0028	0.0000	0.0000
0.0000	0.0000	0.0068	0.0027	0.0028	0.0000	0.0000	0.0000	0.0000	0.0000	0.0027

**Table 4:** Quality evaluation of the variance of deblurred images.

Variance	Point 1	Point 2	Point 3	Dongying	Tianjin
The original image	10.21	9.46	9.37	60.77	67.20
The method used by this paper	11.95	11.35	10.87	64.49	71.98
Method Two	10.38	9.68	9.73	61.88	68.45
Method Three	10.63	10.01	10.17	61.94	70.05

**Table 5:** Quality evaluation of the Tenengrad of deblurred images.

TenenGrad	Point 1	Point 2	Point 3	Dongying	Tianjin
The original image	3.57	2.80	2.91	2517.4	4953.6
The method of this paper	6.76	4.93	4.80	5775	14122
Method Two	4.18	3.18	3.66	3357.1	6696
Method Three	5.18	4.02	4.76	3402.5	7240.4
